# Observations on Sleep-Disordered Breathing in Idiopathic Parkinson’s Disease

**DOI:** 10.1371/journal.pone.0100828

**Published:** 2014-06-26

**Authors:** Philipp O. Valko, Sabrina Hauser, Michael Sommerauer, Esther Werth, Christian R. Baumann

**Affiliations:** Department of Neurology, University Hospital of Zurich, Zurich, Switzerland; The Chinese University of Hong Kong, Hong Kong

## Abstract

**Background:**

This study has two main goals: 1.) to determine the potential influence of dopaminergic drugs on sleep-disordered breathing (SDB) in Parkinson’s disease (PD) and 2.) to elucidate whether NREM and REM sleep differentially impact SDB severity in PD.

**Methods:**

Retrospective clinical and polysomnographic study of 119 consecutive PD patients and comparison with age-, sex- and apnea-hypopnea-index-matched controls.

**Results:**

SDB was diagnosed in 57 PD patients (48%). Apnea-hypopnea index was significantly higher in PD patients with central SDB predominance (n = 7; 39.3±16.7/h) than obstructive SDB predominance (n = 50; 20.9±16.8/h; *p* = 0.003). All PD patients with central SDB predominance appeared to be treated with both levodopa and dopamine agonists, whereas only 56% of those with obstructive SDB predominance were on this combined treatment (*p* = 0.03). In the whole PD group with SDB (n = 57), we observed a significant decrease of apnea-hypopnea index from NREM to REM sleep (*p* = 0.02), while controls revealed the opposite tendency. However, only the PD subgroup with SDB and treatment with dopamine agonists showed this phenomenon, while those without dopamine agonists had a similar NREM/REM pattern as controls.

**Conclusions:**

Our findings suggest an ambiguous impact of dopamine agonists on SDB. Medication with dopamine agonists seems to enhance the risk of central SDB predominance. Loss of normal muscle atonia may be responsible for decreased SDB severity during REM sleep in PD patients with dopamine agonists.

## Introduction

Night-time sleep disturbances are highly prevalent in idiopathic Parkinson’s disease (PD). In contrast to well-established nocturnal phenomena such as rapid eye movement (REM) sleep behaviour disorder (RBD), the prevalence and clinical significance of sleep disordered breathing (SDB) in PD is subject of ongoing controversy. In the last decade, the findings of many groups have consistently pointed to a high prevalence of SDB in PD, ranging from 43–66% [1]–[9]. Two recent studies, however, challenged this view and concluded that SDB is not more common in PD than in age-matched controls [10], [11]. Eventually, emphasizing the absence of characteristic consequences such as increased cardiovascular morbidity and, in particular, the lack of correlation between SDB and excessive daytime sleepiness (EDS), the pathophysiologic profile of SDB in PD has been assumed to differ from that observed in “classical” SDB, and its overall clinical significance has been questioned [5], [10]–[12].

Apart from the discussion on prevalence and clinical significance, several other aspects of SDB in PD remain unclear and shall be addressed in the present study. First, since EDS in PD patients is considered a multi-factorial symptom related to neurodegeneration, sleep-wake disturbances and dopaminergic medication, there is a particular need to define other clinical predictors of SDB in PD. Second, a comparison of clinical and polysomnographic characteristics between PD patients with obstructive and central SDB predominance has not been performed yet. Third, there are contradictory reports on the modulation of respiratory function by antiparkinsonian treatment, but the effect of dopaminergic drugs on SDB remains unknown [13], [14]. Finally, while atonia of upper airway muscles during REM sleep is often associated with increased SDB severity in non-neurological patients, little is known about the differential impact of REM and non-REM (NREM) sleep on SDB in PD [15]. Likewise, the presence of REM-related SDB – a specific subtype of SDB, in which apneas and hypopneas occur predominantly during REM sleep [16]–[19] – has never been assessed in PD.

To answer the abovementioned questions, we retrospectively analyzed clinical and polysomnographic data of 119 consecutive, unselected PD patients. In a second step, we compared the polysomnographic characteristics of PD patients with SDB (PD-SDB) to an age-, gender- and AHI-matched control group with SDB (Co-SDB).

## Methods

### Subjects

This study was conducted at the Department of Neurology of the University Hospital of Zurich, Switzerland. We retrospectively analyzed data of 119 consecutive PD patients who were examined by video-polysomnography (PSG) between January 2005 and June 2010, and in whom motor examinations off medication were available. All patients were examined in our Movement Disorders outpatient clinic and in our sleep laboratory. Due to the high prevalence of sleep-wake disturbances in PD and their possible diagnostic, therapeutic and medico-legal implications, PSG has become an integral diagnostic procedure in our clinic. Thus, our patient group is unselected and representative of a general PD population referred to a tertiary center.

The control group was recruited from a cohort of 200 consecutive non-neurological subjects, who were referred to our sleep lab with suspected SDB. Eventually, we diagnosed SDB in 133 subjects, of whom we included 57 subjects as controls after careful matching for age, sex and apnea-hypopnea index (AHI).

This study was approved by the Ethics Committee of the Canton of Zurich, specialized subcommittee for Psychiatry, Neurology, Neurosurgery, and all patients gave written consent for scientific analyses of their clinical and sleep laboratory results.

### Clinical and polysomnographic assessment

Diagnosis of PD was made according to international diagnostic criteria [20]. The motor subset of the Unified Parkinson’s Disease Rating Scale (UPDRS III) was used for evaluation of motor symptoms severity off medication [21]. All patients underwent detailed structured interviews for the assessment of non-motor symptoms. We assessed sleepiness with the Epworth Sleepiness Scale (ESS), with scores ≥10 indicating excessive daytime sleepiness (EDS) [22]. Furthermore, all patients filled in the Sleep Apnea scale of the Sleep Disorders Questionnaire (SAS-SDQ) which was used as screening tool for SDB [23].

The majority of PD patients were on their usual dopaminergic treatment during sleep laboratory examinations. To compare different dopaminergic medications at doses of equivalent efficacy, we converted all dosages to levodopa dosage equivalents (LDE) according to the following formula: LDE = (regular levodopa dose×1)+(levodopa controlled release dose×0.75)+(pramipexole dose×67)+(ropinirole dose×16.67)+(pergolide dose×100)+(cabergoline dose×50)+(levodopa/entacapone dose×1.25) [24].

We performed PSG recordings from 11.00 PM until 7.00 AM using a 16-channel recording system (Somnologica software; Embla A10, Embla, Broomfield, CO). PSG included electroencephalographic recording (C3-A2, O2-A1), electro-oculography, submental and bilateral anterior tibialis muscles electromyography (EMG), oro-nasal thermistor, nasal flow pressure sensor and measurement of thoracic and abdominal movements (impedance). Oxygen saturation (SaO_2_) was continuously determined with a finger oximeter. Scoring of sleep stages and respiratory events was performed visually using standardized criteria [25]–[27]. Accordingly, REM sleep was scored in the presence of rapid, desynchronized low-voltage EEG, detection of rapid and random movements of eyes, and loss of physiological muscle atonia. REM sleep behavior disorder (RBD) was diagnosed in the presence of increased EMG tone for at least 20% of the total REM sleep duration [27]. For the purpose of this study, RBD was also diagnosed in PD patients showing REM sleep without atonia but without overt dream enacting behavior. Apnea was defined along by a cessation of oro-nasal airflow longer than 10 seconds, and hypopnea by a reduction of oro-nasal airflow by at least 50%, lasting more than 10 seconds and accompanied by an arousal or SaO_2_ reduction of ≥3% [26]. AHI was calculated by the mean number of apneas and hypopneas per hour. SDB was diagnosed in the presence of an AHI≥5/h. SDB severity was classified as mild (AHI: 5–15/h), moderate (AHI: 15–30/h), and severe (AHI≥30/h) [26]. Apneas were obstructive if accompanied with continuous respiratory effort, central if unaccompanied by evidence of respiratory effort, and mixed if a central apnea developed respiratory effort with evidence of obstruction later in the apneic interval. We divided PD patients with SDB in those with obstructive SDB predominance and those with central SDB predominance, if more than 50% of all apnoic events – i.e. the sum of obstructive, mixed and central apneas – were obstructive or central, respectively. In addition, the indices of each type of apneas were calculated separately for NREM sleep and REM sleep. REM-related SDB was defined as an AHI-REM/AHI-NREM ratio >2 [17]. Furthermore, mean SaO_2_, time with SaO_2_ below 90% (SaO_2_<90%), and oxygen desaturation-index (ODI) were calculated.

### Data analysis and statistics

Statistical analyses were performed using SPSS (version 19.0). Group data are described by means and standard deviations (SD). We used the Kolmogorov-Smirnov test to verify whether data were normally distributed. We applied Student’s *t* test for univariate analysis if data were normally distributed, otherwise we used the Mann-Whitney *U* test. Correlation analysis between polysomnographic and clinical variables was calculated with the Spearman’s rank coefficient rho. Group comparison of nominal scale variables was performed with the *χ* ^2^-test. As this study was a purely exploratory study without any specific a priori hypothesis, additional regression analysis with adjustment for multiplicity was not performed. Significance was accepted at *p*<0.05.

## Results

### Prevalence and correlates of SDB in PD

SDB was diagnosed in 57 PD patients (48%), and was mild in 27 (23%), moderate in 14 (12%), and severe in 16 (13%) patients (table 1). Age, sex and disease severity were similar in PD-SDB and PD patients without SDB (PD-wo), and the majority of subjects in both groups suffered from the akinetic-rigid disease type (68%) as opposed to the tremor-dominant PD type (27%). Motor and non-motor Parkinson symptoms including motor fluctuations (37% vs. 39%), hallucinations (28% vs. 28%), REM sleep behaviour disorder (66% vs. 71%), and autonomic disturbances (66% vs. 53%) were equally distributed between PD-SDB and PD-wo. PD-SDB were more often on levodopa (67 vs. 48%, *p* = 0.03) and on dopamine agonists (67 vs. 50%, *p* = 0.049), but total LDE did not differ between PD-wo (551±429) and PD-SDB (595±405; *p* = 0.57). Likewise, when LDE was compared separately for levodopa and dopamine agonists, PD patients with and without SDB had similar LDE for levodopa (449±300 vs. 404±337, *p* = 0.49) and dopamine agonists (194.8±164 vs. 153±198, *p* = 0.11). Eventually, in PD-SDB and PD-wo there was similar use of hypnotics (7% vs. 15%, *p* = 0.25), sedating antidepressants (12% vs. 19%, *p* = 0.33) or activating antidepressants (14% vs. 6%, *p* = 0.23).

**Table 1:** Group comparison of demographic, clinical and polysomnographic characteristics, 1) between PD patients with and without SDB, and 2) between PD patients with SDB and control subjects with SDB.

	PD-wo (n = 62)	PD-SDB (n = 57)	Co-SDB (n = 57)	*p* (PD-wo vs. PD-SDB)	*p* (PD-SDB vs. Co-SDB)
*Demographic data*					
Sex, male	34 (55%)	40 (70%)	40 (70%)	0.06	0.58
Age, y	65±10	66±8	65±8	0.52	0.51
*Clinical characteristics*					
BMI, kg/m^2^	22.9±3.2	26.8±4.5	28.3±5.0	**<0.001**	0.12
Normal BMI (<25)	50 (81%)	21 (37%)	17 (30%)	**<0.001**	0.28
SAS-SDQ-score	28.7±6.2	33.2±7.2	38.7±6.7	**0.002**	**<0.001**
ESS	8.4±4.8	10.0±5.3	11.5±5.1	0.08	0.16
ESS≥10	24/60 (40%)	28/56 (50%)	36/55 (65%)	0.19	0.07
Disease duration, y	8.2±6.1	9.3±6.9		0.41	
UPDRS III	24.3±12.9	26.8±13.2		0.35	
*Polysomnographic findings*					
Total sleep time, min	310±95	323±74	356±79	0.43	**0.02**
Sleep latency to NREM2, min	36±49	29±41	32±39	0.33	0.74
Arousal index, /h	9.5±13.7	10.7±8.4	19.9±19.4	0.22	**<0.001**
Sleep efficiency, %	73±18	74±15	82±14	0.84	**0.006**
Wake, %	26.7±17.7	25.3±15.4	17.5±14.1	0.98	**0.003**
REM, %	11.5±7.0	13.5±7.9	12.8±6.4	0.15	0.15
NREM1, %	11.7±7.3	13.0±8.6	15.7±11.7	0.48	**0.003**
NREM2, %	36.1±13.0	37.1±12.0	38.8±11.6	0.66	0.79
SWS, %	14.1±9.9	11.1±7.9	15.0±7.7	0.07	**0.008**
Apnea-hypopnea index, /h	1.5±1.5	23.2±17.7	23.6±15.7	**<0.001**	0.52
Obstructive apnea index, /h	0.5±0.8	8.5±11.2	10.4±9.4	**<0.001**	**0.03**
Oxygen desaturation index, /h	1.6±1.5	19.0±14.4	21.9±15.5	**<0.001**	0.22
Mean SaO_2_, %	95.0±1.7	94.2±1.8	92.8±2.5	**0.01**	**<0.001**
Minimal SaO_2_, %	89.2±4.0	85.3±6.6	81.4±7.8	**<0.001**	**0.001**
SaO_2_<90%, min	1.8±9.0	9.4±18.1	39.7±84.1	**<0.001**	**0.001**
Apnea mean duration, sec	8.9±8.4	19.5±6.2	19.3±6.4	**<0.001**	0.90

*BMI: body mass index; Co-SDB: controls with sleep-disordered breathing; ESS: Epworth Sleepiness Scale;*

*NREM: non-REM; PD-SDB: Parkinson’s disease patients with sleep-disordered breathing; PD-wo: Parkinson’s disease patients without sleep-disordered breathing; REM: rapid eye movement; SaO_2_: oxygen saturation; SWS: slow wave sleep; SAS-SDQ: Sleep Apnea scale of the Sleep Disorders Questionnaire; UPDRS: Unified Parkinson’s Disease Rating Scale.*

### Clinical predictors of SDB in PD

PD-SDB had higher BMI (*p*<0.001) and SAS-SDQ-scores (*p* = 0.002) than PD-wo (Table 1). The prevalence and severity of EDS was similar in both PD groups (mean ESS values: 10.0±5.3 vs. 8.4±4.8). Subgroup analysis of only male PD patients with BMI ≥25 (n = 33) revealed a prevalence of SDB of 79% (sensitivity 65%, specificity 79%). Polysomnographic findings did not differ between the two groups.

PD-SDB had lower BMI and SAS-SDQ-scores than Co-SDB. Interestingly, the prevalence of excessive daytime sleepiness tended to be higher in Co-SDB than in PD-SDB. Although matched for AHI, PD-SDB had fewer obstructive apneas and less severe indices of oxygen desaturation.

### Characteristics of central SDB predominance in PD

Seven (12%) of the 57 PD-SDB patients had central SDB predominance. Compared to PD patients with obstructive SDB predominance (n = 50), these patients revealed a higher AHI (39.3±16.7 vs. 20.9±16.8, *p* = 0.003). On the other hand, BMI and SAS-SDQ-scores were lower in PD patients with central SDB predominance (table 2). All seven PD patients with central SDB predominance (100%) were treated with both L-Dopa and DA (ropinirole, n = 4; pramipexole, n = 2; cabergoline, n = 1), whereas only 56% of those with obstructive SDB predominance were on this combined treatment (*p* = 0.03). LDE was higher in PD patients with central (684±137) than obstructive SDB predominance (583±429), without however reaching statistical significance (*p* = 0.21). However, while LDE of levodopa did not show differences (475±276 vs. 446±306, *p* = 0.84), LDE of dopamine agonists tended to be higher in PD patients with central SDB predominance (332±162 vs. 178±158, *p* = 0.055). On the other hand, a similar minority in both groups was treated with hypnotics and antidepressants (table 2). Disease duration correlated with central apnea indices (rho = 0.31, *p* = 0.02), but not with obstructive apnea indices (rho = 0.11, *p* = 0.42) or AHI (rho = 0.12, *p* = 0.37). Comparing PD-SDB patients with (n = 38) and without (n = 19) dopamine agonists, the former had a significantly higher central apnea index (4.7/h vs. 0.2/h, *p* = 0.01).

**Table 2:** Comparison of Parkinson’s disease patients with sleep-disordered breathing with predominant central apneas with those with predominant obstructive apneas.

	Central SDB	Obstructive SDB	*p*
	(n = 7)	(n = 50)	
Sex, male	6 (86%)	34 (68%)	0.32
Age, y	70±7	65±8	0.16
BMI, kg/m^2^	24.2±2.7	27.2±4.6	**0.03**
SAS-SDQ-score	26.5±7.8	33.5±7.1	**0.02**
ESS	12.4±6.4	9.7±5.2	0.32
EDS	5 (71%)	23 (47%)	0.21
Disease duration, y	12.7±4.8	8.9±7.0	0.09
UPDRS III	32.6±15.2	26.0±12.9	0.31
L-Dopa	7 (100%)	31 (62%)	**0.048**
Dopamine agonist	7 (100%)	29 (58%)	**0.03**
Both L-Dopa and dopamine agonist	7 (100%)	28 (56%)	**0.03**
LDE (total)	684±137	583±429	0.21
LDE (levodopa)	475±276	446±306	0.84
LDE (dopamine agonists)	332±162	178±158	0.06
Hypnotics	1 (14%)	3 (6%)	0.34
Sedative antidepressants	1 (14%)	6 (12%)	0.57
Activating antidepressants	2 (28%)	6 (12%)	0.15
Total sleep time, min	314±74	324±75	0.77
Sleep latency to NREM2, min	13.9±10.9	31.1±43.3	0.18
Arousal index, /h	9.0±7.0	11.0±8.6	0.52
Sleep efficiency, %	71.0±19.5	75.0±14.9	0.63
Wake, %	29.0±19.5	24.8±14.9	0.61
REM, %	11.6±7.9	13.7±7.9	0.53
NREM1, %	10.7±4.6	13.3±9.0	0.51
NREM2, %	31.7±7.7	37.9±12.4	0.10
Slow wave sleep, %	17.1±11.1	10.2±7.1	0.16
Apnea-hypopnea index, /h	39.3±16.7	20.9±16.8	**0.003**
ODI,/h	32.3±16.7	17.1±13.1	0.06
Mean SaO_2_, %	94.2±1.7	94.2±1.8	0.96
Apnea mean, sec	18.0±2.3	19.7±6.6	0.19

BMI: body mass index; EDS: excessive daytime sleepiness; ESS: Epworth Sleepiness Scale; LDE: levodopa dosage equivalents; L-Dopa: levodopa; NREM: non-REM; REM: rapid eye movement; SaO_2_: oxygen saturation; SAS-SDQ: Sleep Apnea scale of the Sleep Disorders Questionnaire; SDB: sleep-disordered breathing; UPDRS: Unified Parkinson’s Disease Rating Scale.

### Differential impact of NREM and REM sleep on SDB severity

In PD-SDB, obstructive apnea indices (8.4±11.8 vs. 7.2±12.2, *p* = 0.05), central apnea indices (3.6±7.8 vs. 0.5±1.4, *p* = 0.003), and AHI (23.4±19.2 vs. 17.3±19.3, *p* = 0.02) were significantly higher during NREM sleep as compared to REM sleep. Of note, PD patients with central SDB predominance had much higher central apnea indices during NREM sleep than during REM sleep (21.5±9.4 vs. 3.0±3.0, *p* = 0.002), and similarly increased AHI (42.9±17.7 vs. 16.6±20.4, *p* = 0.02), while PD patients with obstructive SDB predominance had a similar AHI during NREM and REM sleep (20.6±17.8 vs. 17.4±19.4, *p* = 0.09). In Co-SDB, on the other hand, obstructive apnea indices and AHI tended to be higher during REM sleep (10.8±17.0 vs. 17.6±19.1, *p* = 0.15, and 21.2±15.8 vs. 28.3±21.8, *p* = 0.15, respectively).

**Figure 1:**
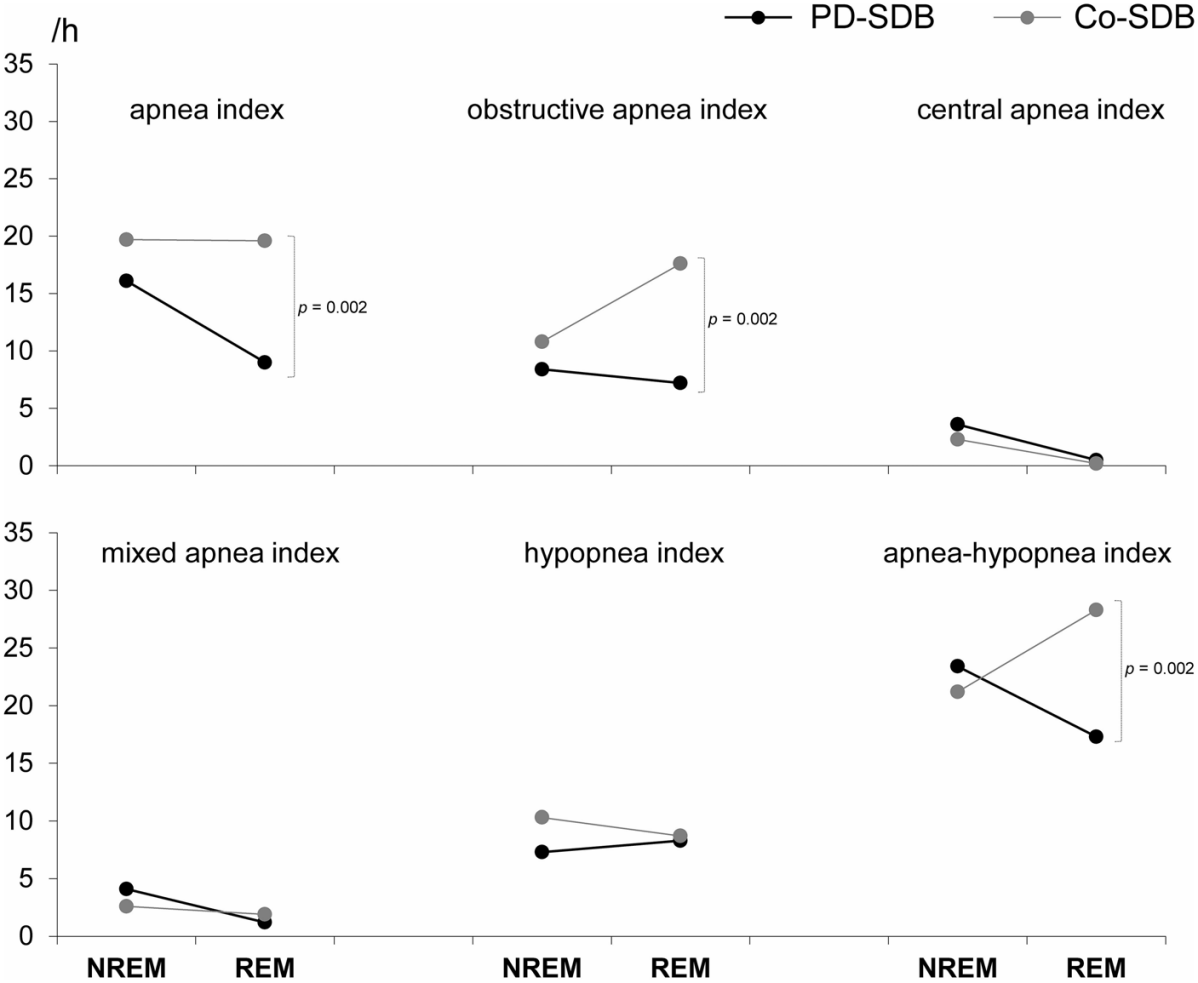
In contrast to controls with sleep-disordered breathing (Co-SDB), SDB severity in Parkinson’s disease (PD) is associated with a significant decrease during REM sleep.

During NREM sleep, we found all apnea indices to be similar between PD-SDB and Co-SDB (Fig. 1). During REM sleep, however, PD-SDB had a significantly lower AHI as Co-SDB (17.3±19.3 vs. 28.3±21.8, *p* = 0.002), which was mainly caused by differences in the obstructive apnea index (7.2±12.2 vs. 17.6±19.1, *p* = 0.001).

### Potential Impact of dopaminergic treatment

**Figure 2:**
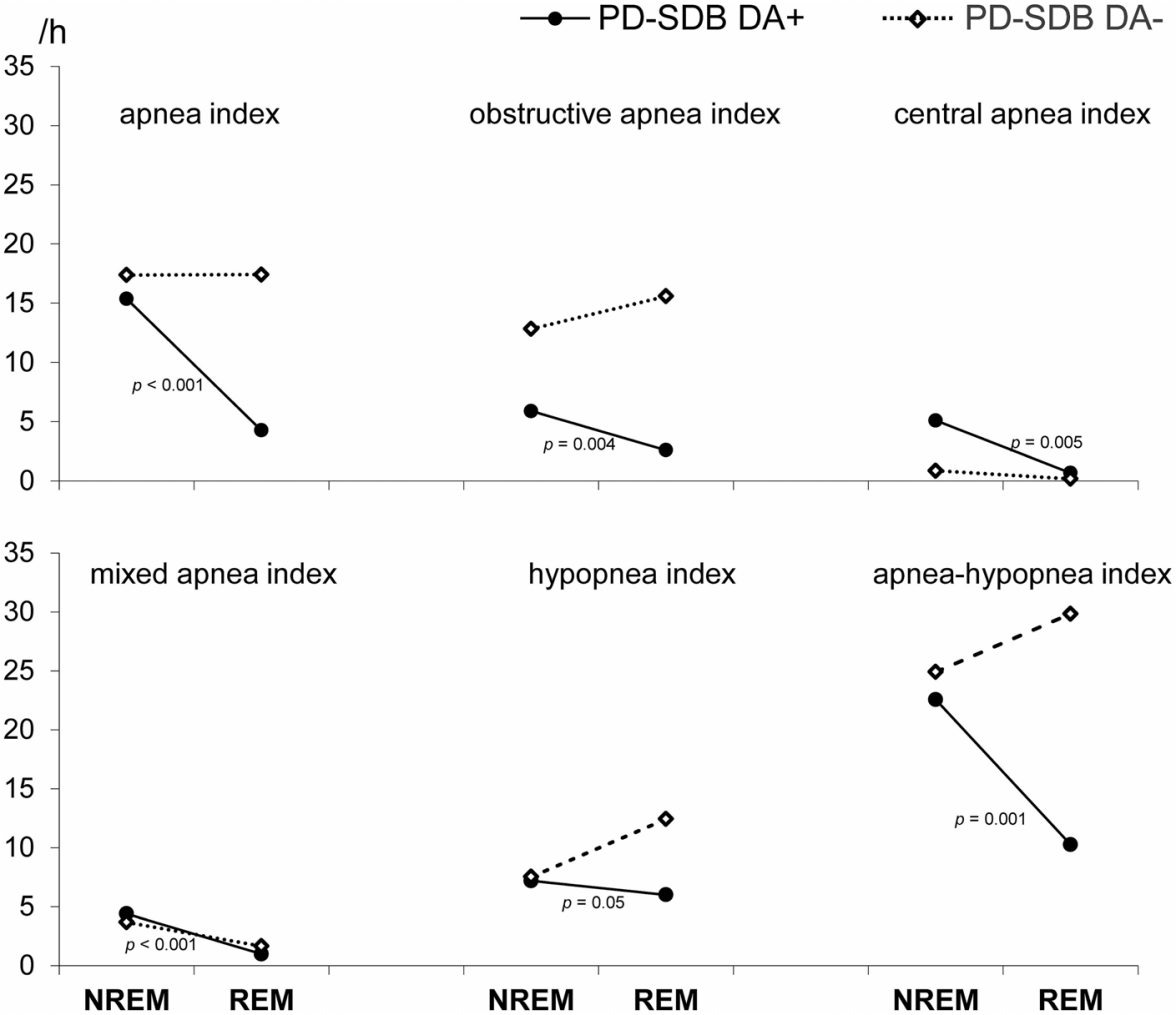
Only PD-SDB patients treated with a dopamine agonist (DA+) showed a significant decrease of SDB severity during REM sleep.

A significant decrease of various apnea indices during REM sleep was only found in PD-SDB treated with a dopamine agonist (PD-SDB/DA+, n = 36) (Fig. 2). PD-SDB/DA+ and PD-SDB/DA- patients did not differ in terms of age, gender, BMI, disease duration, UPDRS III, apnea-hypopnea indices and oxygen desaturation profile, but the former had significantly higher ESS scores (11.8±5.4 vs. 7.1±3.8, *p*<0.001).

### Prevalence and correlates of REM-related SDB in PD

In 8 PD-SDB and in 3 Co-SDB, the presence of REM-related SDB could not be assessed, because they achieved less than 10 min of REM sleep during the total sleep period. SDB severity was high in the excluded subjects, which constitutes a possible reason for the low REM sleep amount (AHI was 37.0±21.5 and 44.8±20.4, respectively). REM-related SDB tended to be less prevalent in PD-SDB than in Co-SDB (22% vs. 37%, *p* = 0.08). In both groups, SDB severity was milder in subjects with predominantly REM-related respiratory events (table 3). In PD patients with REM-related SDB, the prevalence of EDS was significantly lower than in PD patients with not REM-related SDB (9% vs. 62%, *p* = 0.002), and also significantly lower than in PD-wo (*p* = 0.045).

**Table 3:** PD patients and control subjects with and without REM-related SDB.

	PD patients			Control subjects		
	REM-relatedSDB	not REM-relatedSDB	*p*	REM-related-SDB	not REM-relatedSDB	*p*
	(n = 11)	(n = 38)		(n = 20)	(n = 34)	
Sex, male	6 (55%)	28 (74%)	0.20	14 (70%)	25 (74%)	0.51
Age, y	63±10	66±7	0.38	61±7	66±7	**0.02**
BMI, kg/m^2^	30.2±6.1	26.1±3.7	0.05	28.2±3.6	28.5±5.7	0.79
SAS-SDQ-score	31.6±6.7	34.8±6.9	0.20	38.4±6.7	38.8±6.8	0.83
ESS	6.2±2.4	11.5±5.1	**<0.001**	12.3±5.7	10.7±4.6	0.31
ESS≥10	1 (9%)	23 (62%)	**0.002**	15 (75%)	19/32 (59%)	0.20
Disease duration, y	6.1±5.0	10.5±7.2	**0.03**			
UPDRS III	22.4±7.4	27.4±13.4	0.12			
RBD	6 (55%)	29 (81%)	0.09			
Total sleep, min	363±51	331±59	0.09	350±59	373±51	0.15
Sleep latency to NREM2, min	33.8±21.6	23.5±19.5	0.14	21.8±19.1	28.0±30.5	0.36
Arousal index, /h	6.6±5.7	10.7±8.0	0.07	15.1±10.9	22.8±13.2	**0.03**
Sleep efficiency, %	81.8±8.7	76.5±12.0	0.12	81.1±12.8	83.2±11.6	0.55
Wake, %	17.7±9.1	23.4±12.0	0.11	18.9±12.8	15.0±7.9	0.23
REM, %	15.5±5.3	15.0±7.0	0.79	12.3±5.7	12.2±4.4	0.95
NREM1, %	12.2±5.3	13.7±9.8	0.91	14.8±10.4	21.4±14.1	0.06
NREM2, %	42.7±9.6	36.4±11.4	0.08	36.8±10.2	37.3±10.0	0.85
SWS, %	11.8±8.2	11.6±8.1	0.93	17.3±6.2	13.6±7.3	**0.05**
Apnea-hypopjnea index, /h	12.0±4.2	24.8±18.4	0.06	15.9±7.9	26.2±16.5	**0.02**
Apnea index, /h	2.7±2.2	17.5±15.9	**<0.001**	10.0±6.5	17.4±14.1	0.09
Obstructive apnea index, /h	2.3±2.2	9.5±12.0	**0.01**	8.9±5.9	10.4±10.3	0.78
Central apnea index, /h	0.2±0.3	4.2±7.5	0.13	0.3±0.5	3.4±7.8	0.37
Mixed apneaindex, /h	0.2±0.2	4.0±7.7	**0.004**	0.8±1.1	3.4±4.4	**0.01**
Hypopnea index, /h	9.3±4.2	7.3±5.9	0.23	6.0±4.1	9.0±7.7	0.19

*BMI: body mass index; ESS: Epworth Sleepiness Scale; NREM: non-REM; RBD: REM sleep behavior disorder; REM: rapid eye movement; SaO_2_: oxygen saturation; SAS-SDQ: Sleep Apnea scale of the Sleep Disorders Questionnaire; SDB: sleep-disordered breathing; SWS: slow wave sleep UPDRS: Unified Parkinson’s Disease Rating Scale.*

## Discussion

In this retrospective study of 119 consecutive PD patients – so far the largest polysomnographic study on SDB in idiopathic Parkinson’s disease – we found a 48% prevalence of SDB; moderate-severe SDB was diagnosed in 25%. This number is similar to most previous studies on SDB in PD, with reported prevalences ranging from 43–66% [1]–[9]. In comparison, the Sleep Heart Health Study (SHHS) found an AHI≥15/h in 19–21% of 3093 community-dwelling adults aged 60–79 years [28]. Thus, our findings suggest that the prevalence of SDB in PD might not be higher than in the general elderly population.

Compared to other neurological disorders, PD has a particularly high prevalence of EDS. Surprisingly, the prevalence of EDS in our study was not only similar between PD-wo and PD-SDB, but tended to be lower in PD-SDB compared to Co-SDB (50% vs. 65%, *p* = 0.07). Several reasons may explain this observation. First, the contribution of SDB to EDS in PD may be obscured by additional causes, including neurodegenerative disruption of ascending wake-promoting pathways, dopaminergic treatment and the presence of other sleep-wake disturbances. Second, our PD patients have been included in an unselected and therefore referral-unbiased way, whereas EDS was the most frequent complaint prompting sleep studies in Co-SDB. Third, SDB in PD possibly induces less sleep fragmentation, as suggested by a lower arousal index in PD-SDB than in Co-SDB. Finally, although matched for AHI, PD-SDB had fewer obstructive apneas and less severe oxygen desaturation compared to Co-SDB, in other words SDB tended to be milder in PD-SDB. We assessed, however, excessive daytime sleepiness only by the means of questionnaires, which constitutes a limitation because of the possibility that subjective sleepiness in PD patients and controls might be differently estimated.

Due to its high prevalence and complex etiology, EDS is not an appropriate predictor for SDB in PD, nor particularly helpful in identifying those PD-SDB patients who might benefit from CPAP. In accordance with our result, a similar prevalence of EDS between PD patients with and without SDB was demonstrated also by other groups [10], [11]. Therefore, recognition of potential PD-SDB patients should rely on clinical markers other than EDS. Although BMI in PD patients is usually lower than in age-matched healthy controls [29], the findings of our study nevertheless indicate that the diagnosis of SDB should be suspected in PD patients with increased BMI. A pathological BMI (>25) was found in only 19% of PD-wo, while it was common in PD-SDB (63%). Thus, similar to non-neurological SDB patients, male sex and increased BMI represent risk factors of SDB in PD as well. Two previous studies in PD failed to find an association between BMI and SDB [10], [11]. However, the number of PD-SDB patients in these studies was lower (24 and 27, respectively). Furthermore, the patients of the latter study were partly selected by EDS, a selection bias that might have influenced the correlation between BMI and SDB [11]. In addition to BMI, the SAS-SDQ-score may also serve as an appropriate screening tool for SDB in PD patients, as it was found to be significantly higher in PD-SDB compared to PD-wo. Nevertheless, diagnosis of SDB in PD remains challenging and a higher degree of clinical suspicion is needed, as both BMI and SAS-SDQ-score were lower in PD-SDB than in Co-SDB.

On the other hand, BMI and SAS-SDQ-scores did not predict central SDB predominance. AHI was much higher in PD patients with central SDB predominance than in those with obstructive SDB predominance, but the former had nevertheless significantly lower BMI and SAS-SDQ-scores. Conversely, central SDB predominance might be suspected in every PD patient with longer disease duration and, in particular, dopaminergic treatment consisting of both L-Dopa and a dopamine agonist. Our results, however, must be interpreted with caution because of the low number of PD patients with central SDB predominance. An association between the use of dopamine agonists and sudden sleep attacks has been reported by several groups [30]–[33]. None of these studies, however, has searched for SDB. Our results indicate that central SDB predominance might contribute to sleep attacks in PD patients treated with dopamine agonists. In this line, the prevalence of EDS was particularly high in our PD patients with central SDB predominance (71%). The possible causal relationship between DA treatment and central SDB predominance on one hand and sleep attacks on the other hand, highlights the need to better elucidate subgroups of PD-SDB, in whom CPAP treatment may be indicated. Response to CPAP treatment could provide some insights on whether EDS in PD patients with central SDB predominance is primarily caused by the dopaminergic treatment or by SDB. Thus, future studies should include higher numbers of well-characterized patients to confirm or reject our finding. Whether factors other than dopaminergic medication, namely disease duration and more advanced disease stage, are more likely to contribute to central SDB predominance remains uncertain at this moment, but impaired central respiratory regulation due to diffuse brainstem degeneration is certainly another risk factor for increased central SDB predominance.

In PD, SDB severity significantly decreased during REM sleep, while in controls SDB was more likely to increase during REM sleep. Likewise, the prevalence of REM-related SDB tended to be lower in PD compared to controls. In other words, REM sleep in PD is associated with decreased SDB severity. These findings support our initial hypothesis and indicate that frequent incomplete REM atonia in PD might counterbalance the REM-associated tendency of the upper airway to collapse. In the same line, only OAI tended to increase during REM sleep in Co-SDB, while the remaining apnea indices remained stable. However, many PD patients still suffered from SDB despite the presence of polysomnographically proven RBD, and the prevalence of RBD did not differ between PD-wo and PD-SDB. This observation was also made by Cochen De Cock et al, who even reported a higher REM AHI in PD patients with abnormal persistence of chin muscle tone compared to those with normal REM atonia [11].

On the other hand, a major finding of our study is the striking effect of dopamine agonists on REM-associated SDB severity. AHI reduction during REM sleep was only found in PD-SDB treated with a dopamine agonist, while PD-SDB without dopamine agonists showed a similar NREM/REM pattern as Co-SDB. Taken together, both intrinsic factors related to neurodegeneration of REM sleep modulating brainstem structures and extrinsic, drug induced factors probably account for the observed decrease SDB severity during REM sleep.

Finally, only 9% of PD patients with REM-related SDB had EDS, a remarkably low number when considering the high prevalence of EDS in PD. In this line, two previous, multiple sleep latency test (MSLT) based studies found that only respiratory events during NREM sleep contributed to increased daytime sleepiness [34], [35]. However, a decreased impact of REM-related respiratory events on daytime sleepiness does not explain why PD patients with REM-related SDB suffer significantly less often from EDS than PD-wo. Alternatively, one might speculate that many of the brainstem structures involved in REM sleep regulation – such as the noradrenergic locus coeruleus, the serotonergic dorsal raphe and the cholinergic pedunculopontine nucleus – are more preserved in this specific PD-SDB subpopulation. Since these neurotransmitters represent major components of the ascending wake-promoting system, their integrity might explain the low prevalence of EDS.

We have to acknowledge several limitations. First of all, the study is retrospective. This allowed including a relatively large number of PD patients, but larger studies are required to verify our findings. In the same line, our exploratory study did not include multiple regression models due to the lack of specific a priori hypotheses. Second, the recruitment procedure for PD patients and control subjects was different. In PD patients, a referral bias seems unlikely, because polysomnography was performed in most PD patients independent of the presence or absence of (suspected) SDB. While this is clearly a strength of our study, the inclusion criteria of the control subjects were quite different, as they were all referred with the suspicion of SDB. Finally, our observations cannot be generalized, as we examined only PD patients seen in a tertiary care center, possibly representing a more advanced and complicated disease course. In such patients, polypharmacy is probably more common, which might have caused more central apneas.

In conclusion, SDB in PD is frequent but its clinical diagnosis is more challenging, because both risk factors and consequences are less evident. Dopaminergic treatment, namely dopamine agonists, seems to have an ambiguous influence on SDB, as it may simultaneously contribute to severe central SDB predominance on one hand, and lessen REM associated SDB severity on the other hand. Future CPAP trials may be meaningful in better characterising those PD-SDB patients, who will benefit in terms of EDS and sleep attacks.
